# Postnatal hospitalization and self-esteem in mothers

**DOI:** 10.1192/j.eurpsy.2023.1515

**Published:** 2023-07-19

**Authors:** K. Chiha, K. Khemakhem, C. Regaieg, D. Ben Touhemi, H. Ayadi, N. Hmida, A. Gargouri, Y. Moalla

**Affiliations:** 1 Child Psychiatry; 2 Neonatology Department, Hedi Chaker University Hospital, Sfax, Tunisia

## Abstract

**Introduction:**

Hospitalization in neonatology constitutes a narcissistic wound for the mother. This mother-baby separation disturbs the maternal identity and generates a strong feeling of failure and guilt.

**Objectives:**

To assess self-esteem in mothers of babies hospitalised during the postnatal period in the neonatal unit and to identify risk factors associated with the persistence of low self-esteem 3 months after discharge.

**Methods:**

This was a longitudinal, descriptive and analytical study conducted between April and September 2021. The sample consisted of mothers of babies hospitalized in the neonatology department of Sfax-Tunisia for a period ranging from 5 to 15 days. Socio-demographic data were collected using a pre-established form. Self-esteem was assessed during the baby’s hospitalisation and 3 months after discharge, using the Rosenberg self-esteem scale, with 10 items, validated in Arabic.

**Results:**

The sample consisted of 86 mothers with a mean age of 32.17 years.

Low to very low self-esteem was found in mothers in 81.20% of cases when their babies were hospitalised and in 68.40% of cases 3 months after discharge.

Some factors were significantly related to the persistence of low self-esteem in mothers after 3 months of their babies’ hospitalisation, such as a low educational level of the mother (p=0.017), an unattended pregnancy (p=0.034), the occurrence of a post-partum complication (p=0.043) and the absence of the first smile in the baby at the age of 3 months (p=0.008).

**Conclusions:**

This study shows a high prevalence of low self-esteem in mothers following hospitalization of their babies in the postnatal period. The concomitance with several socio-clinical factors contributes to the persistence of this low level of self-esteem in these mothers.

Appropriate early and multidisciplinary care helps to rebuild strong self-esteem in the young mother so that she can overcome her psychological difficulties and build a solid foundation for the mother-baby bond.

**Disclosure of Interest:**

None Declared

